# Hausärztliche Medizin im digitalen Zeitalter: Szenarien und Empfehlungen

**DOI:** 10.1055/a-1791-0834

**Published:** 2022-07-14

**Authors:** Kristina Spöhrer, Eike Buhr, Andreas Hein, Mark Schweda, Martin Scherer

**Affiliations:** 1 Institut und Poliklinik für Allgemeinmedizin, Universitatsklinikum Hamburg-Eppendorf, Hamburg, Germany; 2 Ethik in der Medizin, Department für Versorgungsforschung, Fakultät VI – Medizin und Gesundheitswissenschaften, Carl von Ossietzky Universität Oldenburg, Oldenburg, Germany; 3 Assistenzsysteme und Medizintechnik, Department für Versorgungsforschung, Fakultät VI – Medizin und Gesundheitswissenschaften, Carl von Ossietzky Universität Oldenburg, Oldenburg, Germany

**Keywords:** Hausärztliche Medizin, Szenario-Technik, Digitalisierung, Zukunftsszenario, Family Medicine, Scenario Method, Digitalization, Future Scenario

## Abstract

**Ziel** Der Beitrag nimmt die Situation der hausärztlichen Medizin im digitalen Zeitalter in den Blick, um künftige Entwicklungsperspektiven zu erörtern und Handlungsmöglichkeiten aufzuzeigen.

**Methodik** Es wurde ein strukturierter Verständigungsprozess unter Einbeziehung relevanter Stakeholder-Perspektiven durchgeführt, der sich konzeptioneller Ansätze und methodischer Elemente der Szenario-Technik bediente. Diese Technik ermöglicht auf der Grundlage einer empirisch informierten Analyse gegenwärtiger Sachlagen und Entwicklungstrends die Formulierung von Zukunftsszenarien und die Ableitung praktischer Handlungsempfehlungen.

**Ergebnisse** In Verlängerung gegenwärtiger Tendenzen im Bereich der Ärzteschaft, der Patientinnen und Patienten, der technischen Entwicklung und des Gesundheitswesens wurden ein Best- und ein Worst-Case-Szenario zur hausärztlichen Medizin im Jahr 2050 entwickelt. Aus der Analyse und Erörterung der Szenarien ließen sich Handlungsempfehlungen für die Gegenwart ableiten.

**Schlussfolgerung** Ausgehend von den entwickelten Szenarien formulieren wir zwölf Empfehlungen zur künftigen Stärkung einer hausärztlich-zentrierten Medizin, die eine flächendeckende, digital unterstützte holistisch-patientenorientierte Gesundheitsversorgung ermöglicht.

## Abstract (en)

**Aims** The aim of this study was to examine the situation of family medicine in the digital age in order to discuss future trends and outline recommendations for action.

**Methods** We conducted a structured deliberative process employing elements of the scenario method and involving relevant stakeholder perspectives. Based on an empirically informed analysis of current situations and trends, the scenario method allows the formation of practical recommendations.

**Results** Extrapolating current trends in the medical profession, the patients, the technological development, and the healthcare system, we developed a best case and a worst case scenario of family medicine in the year 2050. From the analysis and discussion of the scenarios, we derived recommendations for practitioners and decision makers.

**Conclusions** Based on the developed scenarios, we recommend twelve measures towards a model of future healthcare that is centered on family medicine and enables a comprehensive, digitally supported holistic and patient-oriented service provision.

## Einleitung

Die hausärztliche Medizin steht gegenwärtig an einem kritischen Punkt. Wenngleich die Versorgung in den meisten Regionen noch sichergestellt werden kann, zeichnen sich schon jetzt drohende Versorgungslücken ab – insbesondere im ländlichen Bereich. Diese Lücken entweder zu verhindern oder zu schließen ist eines der Kernanliegen politischen Handelns.

Der Digitalisierung wird dabei eine wichtige Bedeutung zugeschrieben. Allgemein bezeichnet der Begriff die zunehmende Integration digitaler Technologien in verschiedenen gesellschaftlichen Teilbereichen und Handlungszusammenhängen. In Medizin- und Gesundheitswesen findet Digitalisierung auf allen Ebenen statt, sowohl der Mikro- als auch der Meso- und der Makroebene 1 . Technische Entwicklungen an der Schnittstelle zwischen persönlichen digitalen Assistenztechnologien (z. B. Gesundheitsinformationen über das Internet, Consumer Electronics, Smartphones und Wearables wie Smartwatches, personenbezogene elektronische Gesundheitsakte, Sprach- und Roboter-Assistenten) und den Einrichtungen der Gesundheitsversorgung – insbesondere der Hausarztpraxis (z. B. elektronische Patientenakte, digitale Abrechnung und Rezepte, digitale Diagnostik, Entscheidungsunterstützungssysteme) – können die Entwicklung der nächsten Dekaden und damit die Gesundheitsversorgung der Zukunft maßgeblich prägen.

Vor diesem Hintergrund wird eine möglichst breit geführte Verständigung über Bedingungen und Zielsetzungen guter hausärztlicher Medizin im Zeitalter der Digitalisierung notwendig. Dabei sollte es darum gehen, die allgemeinmedizinisch-hausärztlichen Errungenschaften der letzten Jahrzehnte zu bewahren und auch zur Grundlage der hausärztlichen Versorgung der Zukunft zu machen. Die fortschreitende Digitalisierung ist überwiegend (aber nicht nur) technologiegetrieben und hat das disruptive Potential, bestehende Herangehensweisen grundlegend in Frage zu stellen, zu marginalisieren oder am Ende gar zu ersetzen, so wie das Online-Buchungsportal das frühere Reisebüro. Deshalb erscheint es unerlässlich, dass Ärztinnen und Ärzte sich mit Chancen und Grenzen der digitalen Transformation in Medizin und Gesundheitsversorgung sowie den zu erwartenden Veränderungen aufseiten der Patientinnen und Patienten, der technologischen Entwicklung sowie des Gesundheitswesens auseinandersetzen 2 3 4 .

Diese Ausgangslage gab einer interdisziplinär zusammengesetzten Arbeitsgruppe von Wissenschaftlerinnen und Wissenschaftlern, Professionsvertreterinnen und -vertretern sowie Stakeholderinnen und Stakeholdern den Anstoß zur Durchführung eines strukturierten Verständigungsprozesses über die Zukunft der hausärztlichen Medizin im digitalen Zeitalter. Dabei ging es keinswegs nur um eine isolierte Betrachtung der Digitalisierung und ihrer technischen Chancen und Risiken. Ziel war es vielmehr, zentrale Aspekte und Tendenzen der gegenwärtigen Situation der hausärztlichen Medizin im Zeichen der digitalen Transformation in einem methodisch geregelten Verfahren zu bündeln, zu ordnen und zu evaluieren, um Klarheit und Orientierung im Hinblick auf künftige Entwicklungsperspektiven und Handlungsmöglichkeiten zu gewinnen. Beteiligt waren Vertreterinnen und Vertreter der Allgemeinmedizin, der Medizintechnik/-informatik, der Medizinethik, der Medizinischen Fachangestellten und der Selbsthilfe. Wir hoffen, dass von den im Folgenden vorgestellten Ergebnissen und Empfehlungen dieses Verständigungsprozesses Anregungen für die weitere Auseinandersetzung mit der Zukunft der hausärztlichen Medizin in Deutschland ausgehen.

## Hintergrund und Methode

Um die künftige Entwicklung der hausärztlichen Medizin unter Einbeziehung relevanter Stakeholder-Perspektiven eingehender zu erörtern, wurden konzeptionelle Ansätze und methodische Elemente der so genannten Szenario-Technik genutzt. Dabei handelt es sich um eine Methode strategischer Planung, die ursprünglich aus dem Militärwesen, der Unternehmensführung und der Politikberatung stammt, inzwischen aber auch im wissenschaftlichen Bereich weithin Verwendung findet, etwa in Zukunftsforschung und Technikfolgenabschätzung 5 .

Ziel des Ansatzes ist die systematische Entwicklung und Bewertung von Zukunftsszenarien, auf deren Grundlage sich praktische Handlungsempfehlungen formulieren lassen. Solche Szenarien unterscheiden sich dadurch von bloßen Visionen oder Wunschvorstellungen, dass sie sich auf eine empirisch begründete und methodisch geregelte Analyse gegenwärtiger Sachlagen und Entwicklungstrends stützen und so eine gewisse Plausibilität für sich beanspruchen. Dabei folgt das Vorgehen in der Regel einer Reihe aufeinander aufbauender Schritte (vgl. zum Folgenden etwa 6 ). In einem ersten Schritt wird eine Aufgaben- und Problemanalyse vorgenommen. Dabei ist zunächst der konkrete Gegenstand zu bestimmen, dessen Zukunft betrachtet werden soll. Zudem werden die Faktoren ermittelt, die einen Einfluss auf seine Entwicklung ausüben. In einem weiteren Schritt werden die Stärke und die Wechselwirkung der betreffenden Faktoren bestimmt. Im dritten Schritt werden davon ausgehend unterschiedliche Entwicklungsmöglichkeiten entworfen, die sich in hypothetischen Zukunftsszenarien niederschlagen, beispielsweise einem Trendszenario oder einem Best- bzw. Worst-Case-Szenario. Diese Szenarien werden abschließend erörtert und bewertet, um mit Blick auf die relevanten Einflussfaktoren und Entwicklungstrends geeignete praktische Maßnahmen abzuleiten und Handlungsempfehlungen zu formulieren.

In Anlehnung an die skizzierte Vorgehensweise wurden in dem hier beschriebenen Verfahren einzelne Elemente der Szenario-Technik zur Strukturierung der gemeinsamen Verständigung über die hausärztliche Medizin im digitalen Zeitalter aufgegriffen (vgl. für ein ähnliches Vorgehen 7 ). Aufgrund praktischer und zeitlicher Beschränkungen wurden dabei einzelne Schritte der Problemanalyse, der Bestimmung der Stärke und Wechselwirkung von Einflussfaktoren sowie der Entwicklung von Szenarien ausgespart oder zusammengefasst. Der Kreis der Autorinnen und Autoren sowie der weiteren Mitwirkenden umfasste Vertreterinnen und Vertreter unterschiedlicher (professioneller) Stakeholdergruppen und wissenschaftlicher Fachbereiche und erlaubte so eine multiperspektivische und interdisziplinäre bzw. interprofessionelle Auseinandersetzung (Allgemeinmedizin, Medizintechnik/-informatik, Medizinethik, Medizinische Fachangestellte und Patientenvertretung/Selbsthilfe) Die leitende Fragestellung lautete: *Wie sieht die hausärztliche Medizin in Deutschland im Jahr 2050 aus?*

In einem ersten Schritt wurde eine Verständigung über vorab gesammelte und gruppierte Einflussfaktoren erzielt. Dabei wurden vier wesentliche Einflussbereiche identifiziert, die für die künftige Entwicklung der hausärztlichen Medizin in Deutschland von entscheidender Bedeutung sind: die Ärzteschaft, die Patientinnen und Patienten, die technische Entwicklung und das Gesundheitswesen. Jeder der Bereiche umfasste unterschiedliche Einflussfaktoren, etwa das Selbstverständnis und den gesellschaftlichen Stellenwert der ärztlichen Profession, die soziodemographische Zusammensetzung der Bevölkerung, den Zugang zu und Austausch von Forschungs- und Versorgungsdaten sowie die Verfassung des solidarisch getragenen Gesundheitssystems. Ausgehend von diesen Faktoren und ihrer möglichen weiteren Entwicklung wurden in einem zweiten Schritt begründete Annahmen zur Lage der hausärztlichen Medizin im Jahr 2050 formuliert. Dazu wurde in Verlängerung gegenwärtiger Tendenzen sowohl eine möglichst günstige als auch eine möglichst ungünstige Entwicklung entworfen und in einem Best- bzw. einem Worst-Case-Szenario zusammengefasst. Beide Szenarien wurden anschließend in einem dritten Schritt gemeinsam erörtert, um daraus schließlich Handlungsempfehlungen für die Gegenwart abzuleiten. Dazu haben wir auf der Grundlage der jeweils relevanten Einflussfaktoren analysiert, durch welche Schritte sich der Eintritt des Best-Case Szenarios wahrscheinlicher und der des Worst-Case-Szenarios unwahrscheinlicher machen ließe. Abschließend wurden die relevanten Adressaten und Maßnahmen identifiziert und entsprechende Empfehlungen ausformuliert.

## Ergebnisse

Im Folgenden werden die beiden entworfenen Szenarien zur Entwicklung der hausärztlichen Medizin in Deutschland im Jahr 2050 in ihren wesentlichen Grundzügen umrissen. Im Anschluss sind die daraus abgeleiteten Empfehlungen aufgeführt.

### Best-Case-Szenario

Im Jahr 2050 wird die primäre Gesundheitsversorgung in Deutschland eine hausärztlich zentrierte, holistisch-patientenorientierte Versorgung sein, bei der die Hausarztpraxisteams eine umfassende Verantwortung für ihre Patientinnen und Patienten übernehmen, die auf einem langfristig aufgebauten persönlichen Vertrauensverhältnis basiert. Diese Versorgung wird wesentlich durch hausärztlich geleitete multiprofessionelle Teams erbracht und bei Bedarf durch andere Versorgungsebenen ergänzt. Mit Hilfe rechtlicher, organisatorischer und technischer Innovationen kann so eine inklusive, flächendeckende und kontinuierliche medizinische Grundversorgung unterschiedlicher Bevölkerungsgruppen gewährleistet werden.

Mit Blick auf die Rolle der *Ärztinnen und Ärzte* heißt das, dass die hausärztliche Praxis die erste Anlaufstelle für Menschen mit gesundheitlichen Problemen und Sorgen darstellt. Anstelle eines einzelnen Hausarztes bzw. einer Hausärztin wird diese Praxis aus einem hausärztlich geleiteten Team bestehen. Dieses Team ist multiprofessionell und schließt sowohl mehrere Hausärztinnen bzw. -ärzte als auch Verwaltungs- und Medizinische Fachangestellte sowie z. B. Fachkräfte aus Pflege, Physiotherapie, Psychotherapie und Sozialer Arbeit mit ein. Eine effiziente Aufgabenteilung durch Delegation bisher ärztlicher Leistungen an nicht-ärztliche Gesundheitsfachberufe mit entsprechender Qualifikation wird unverzichtbar, um den steigenden Behandlungsbedarf der alternden Bevölkerung zu bewältigen. Hausärztliche Teams kooperieren zudem mit stationären Einrichtungen (Informationsaustausch und Teilung der diagnostischen und therapeutischen Leistungen) und dem gebietsfachärztlichen Versorgungsbereich (z. B. über digitale Konsile, gezielte Überweisungen, Übernahme von Auftragsleistungen). Darüber hinaus versorgen sie Patientinnen und Patienten bei Bedarf telemedizinisch. Auf diese Weise können zum einen die Hausärztinnen und Hausärzte selbst finanziell, personell und zeitlich entlastet und zum anderen auch psychosoziale Aspekte im Sinne einer holistischen Versorgung kompetent berücksichtigt werden. Solche hausärztlichen Teams könnten eine Zentralisierung bedeuten, aber auch die Abstellung von Personen für einen mobilen Dienst bzw. die ambulante Versorgung in der Fläche ermöglichen (Hausbesuche). Selbsthilfegruppen und ihre Organisationen werden von den Hausärztinnen und Hausärzten als Partner akzeptiert und unterstützt.

*Patientinnen und Patienten* erhalten unabhängig von ihren konkreten Lebensumständen und ihrem jeweiligen Wohnort sowohl in der Stadt als auch auf dem Land rund um die Uhr eine bedarfsgerechte gesundheitliche Grundversorgung. Insbesondere ältere Menschen müssen nicht zu einer entfernten städtischen Hausarztpraxis pendeln, sondern können auch zu Hause oder in Zweigpraxen/mobilen Einheiten vor Ort angemessen versorgt werden. Die medizinische Behandlung durch einen Arzt bzw. eine Ärztin bleibt ungeachtet der multiprofessionellen Arbeitsteilung und technischen Optimierung einzelner Aspekte der Versorgung stets gewährleistet. Eine transparente und niederschwellig erreichbare digitale Infrastruktur trägt zu einer höheren Gesundheitskompetenz und einer Stärkung des Shared Decision Making bei: Auf der einen Seite erhalten Patientinnen und Patienten Informationen über Angebote und Kompetenzen der einzelnen Hausärztinnen und Hausärzte, die sie dann fundiert auswählen können. Auf der anderen Seite sind sie durch die Multiprofessionalität der hausärztlichen Teams und die Berücksichtigung ihrer sozialen Einbettung sowie des biopsychosozialen Modells besser über Möglichkeiten ihres individuellen Gesundheitsverhaltens informiert. Die Selbsthilfe hat sich weiter etabliert, ist in die digitale Infrastruktur integriert und wird dauerhaft öffentlich gefördert. Sie bietet Mitgliedern und Angehörigen psychosoziale Unterstützung und Hilfe zur Selbsthilfe sowie Erfahrungs- und Informationsaustausch.

Unterstützt wird diese Art der Versorgung insbesondere durch die *Digitale Transformation* . Sie stellt moderne, KI-basierte Technik bereit, die eine effizientere Organisation und Verwaltung der hausärztlichen Arbeit ermöglicht und damit zeitliche und personelle Ressourcen für den Kernbereich der Patientenversorgung freisetzt. So können alle Patientinnen und Patienten über Portale oder Chatbots rund um die Uhr erste Einschätzungen zur Kritikalität einer Situation oder der Dringlichkeit einer Versorgung erhalten. Durch den sicheren und verantwortungsvollen Zugriff auf die patienten-individuelle Gesundheitsakte sowie eine große Menge medizinischer Daten sind diese digitalen Portale in der Lage, medizinische Anliegen einzuordnen und umgehend entweder eine Ärztin bzw. einen Arzt zu alarmieren, einen telemedizinischen oder persönlichen Termin in der Hausarztpraxis zu einem gegebenen Zeitpunkt zu vereinbaren oder ein Rezept auszustellen. Fragen des Datenschutzes und der Datenhoheit gewinnen damit an Bedeutung. Ein zentrales Datenmanagementsystem ermöglicht eine zuverlässige Wartung und Supervision und damit auch eine zeitliche Entlastung der Ärztinnen und Ärzte, ist aber auch anfälliger für Manipulation oder Datendiebstahl und -missbrauch als ein dezentrales System. Hier muss unter Beteiligung der relevanten Stakeholder eine Abwägung des Für und Wider unterschiedlicher Ansätze erfolgen.

Auf der Ebene des *Gesundheitssystems* wird die Autonomie und systemisch zentrale Rolle der Hausärztinnen und Hausärzte gestärkt. Dies wird unter anderem über eine Entbürokratisierung der Rahmenbedingungen ihrer Tätigkeiten erreicht. Speziell der Einfluss häufig wechselnder gesetzlicher Regelungen und politischer Reformen sowie die Bedeutung der Regularien der Krankenversicherungen, insbesondere im Hinblick auf die Leistungsabrechnung, werden begrenzt. Insgesamt wird das Gesundheitssystem von einer breiten gesellschaftlichen Verständigung und der gemeinsamen Vision eines gesamtgesellschaftlichen Versorgungsauftrags getragen. Im Rahmen eines solchen Systems wird auch die angemessene Finanzierung einer entsprechenden flächendeckenden gesundheitlichen Grundversorgung und -sicherung gewährleistet.

### Worst-Case-Szenario

Im Jahr 2050 wird die Gesundheitsversorgung in Deutschland stark fragmentiert sein. Partikulare Stakeholder-Perspektiven, kommerzielle Profitinteressen und die Deckelung der Gesundheitskosten prägen die Landschaft. Hausärztinnen und Hausärzte werden dabei keine zentrale Rolle mehr spielen, sondern lediglich ein Rädchen im Getriebe eines weitgehend globalisierten und technisierten Gesundheitsmarktes mit sehr eingeschränkten nationalen Kontrollmöglichkeiten sein. Die Versorgung von Patientinnen und Patienten wird dabei stark von deren unterschiedlichen Lebenslagen, Informationsgraden und finanziellen Situationen abhängen.

Die *Hausärztinnen und Hausärzte* sind im Jahr 2050 keine persönlichen Ansprechpartner mehr, denen eine besondere Rolle oder Steuerungsfunktion in der Gesundheitsversorgung zukommt. Ihre Expertise und ihre Zuständigkeiten sind weitgehend auf verschiedene funktional differenzierte Assistenzberufe übergegangen oder werden durch spezifische technische Anwendungen übernommen. Sie stehen zudem im Wettbewerb mit einer Vielzahl unterschiedlicher, teils international agierender kommerzieller Anbieterinnen und Anbieter von (vielfach fragwürdigen) alternativen Behandlungen und Gesundheitsdienstleistungen. Der Arztberuf ist letztlich ein Dienstleistungsberuf wie jeder andere und eine langfristige Patientenbindung, ein geteiltes Verständnis der Profession sowie eine gemeinsame Vertretung ihrer Angelegenheiten sind weitgehend verschwunden. Eine gesamtgesellschaftliche Verantwortung im Sinne einer Zuständigkeit für die Gesundheit der Bevölkerung wird nicht mehr wahrgenommen.

Für die *Patientinnen und Patienten* bedeutet das, dass sich ihre Versorgungssituation grundlegend verschlechtert. Aufgrund eines Versorgungsgefälles zwischen Stadt und Land und der Abhängigkeit der Gesundheitsversorgung vom individuellen Finanzaufkommen verlieren sie ihre Wahlfreiheit im Hinblick auf die sie behandelnden Ärztinnen bzw. Ärzte. Es kommt zu einer Zwei-Klassen-Medizin mit verstärkter Über-, Unter- und Fehlversorgung. Das Vertrauen in die eigene Ärztin bzw. den eigenen Arzt und eine langfristige, vertrauensbasierte Arzt-Patienten-Beziehung gehen verloren. Stattdessen setzen die Patientinnen und Patienten zunehmend auf internationale Telemedizinangebote, technische Gesundheitsapplikationen mit unklarer Qualitätssicherung sowie Datennutzung und/oder wenden sich zunehmend alternativen Behandlungsmethoden zu. Insgesamt verschlechtert sich ihre Gesundheitskompetenz, das heißt ihre Fähigkeit zur eigenverantwortlichen Wahrnehmung ihrer gesundheitlichen Belange, in einem komplexen (Über-)Angebot von Dienstleistungen. Es überwiegt ein mechanistisch und monodisziplinär geprägtes Medizinverständnis, während der Wunsch nach ganzheitlicher Versorgung multiple komplementärmedizinische Angebote entstehen lässt. Auf diese Weise verlieren sie letzten Endes die Möglichkeit zur informierten Entscheidung über ihre Gesundheitsversorgung. Die Selbsthilfeförderung wird gekürzt und viele Selbsthilfegruppen und ihre Organisationen lösen sich auf. Es bleiben Patientenorganisationen übrig, die von internationalen Konzernen der Gesundheitswirtschaft (wie zum Beispiel Pharmakonzernen) finanziert werden und eng mit ihnen kooperieren.

Die *digitale Transformation* im Gesundheitsbereich wird in erster Linie von marktwirtschaftlichen Kräften vorangetrieben und entfaltet sich eigengesetzlich. Dabei wird der marktwirtschaftliche Wettbewerb kaum durch nationalstaatliche oder internationale Qualitätskriterien oder gesetzliche Regelungen eingeschränkt. Global agierende Wirtschaftsunternehmen tragen über Grenzen hinweg große Mengen an Gesundheitsdaten zusammen, die sie unter kommerziellen Gesichtspunkten auswerten und nutzen. Der Schutz von personenbezogenen Daten spielt dabei allenfalls eine untergeordnete Rolle. Insgesamt herrscht eine große Abhängigkeit von automatisierter Technik und eine Intransparenz hinsichtlich der Zielsetzungen und Verantwortlichkeiten ihrer Verwendung.

Das *Gesundheitssystem* ist in diesem Szenario geprägt von starker staatlicher Lenkung auf der einen und einem radikal marktwirtschaftlichen globalen Gesundheitsmarkt auf der anderen Seite. So sind die Ärztinnen und Ärzte strengen staatlichen Regulierungen sowie starren Abrechnungsmodalitäten der Krankenversicherungen unterworfen, müssen sich aber gleichzeitig auf einem hochgradig kompetitiven Gesundheitsmarkt behaupten. Der verbliebene Handlungsspielraum der Ärztinnen und Ärzte wird also letztendlich von einem ökonomischen Imperativ diktiert. Das Gesundheitssystem folgt hierbei keiner gesamtgesellschaftlichen Vision, die digitale Transformation beschleunigt diese Prozesse und verstärkt partikulare Interessen und marktwirtschaftliche Verwertungslogiken.

### Schluss und Empfehlungen

In der Auseinandersetzung mit diesen beiden Szenarien zeichnen sich Konturen einer möglichen hausärztlichen Medizin im digitalen Zeitalter ab. Zu ihnen gehört wesentlich eine zugleich innovationsoffene und -kritische Grundhaltung, die dem Patientennutzen die oberste Priorität beimisst und eine solide Evaluation neuer, bislang nicht erprobter Verfahren verlangt. Es handelt sich um eine Medizin, die die Spezialisierung auf den ganzen Menschen in den Mittelpunkt stellt. Diese holistische Patientenversorgung, die derzeit schon tagtäglich in Hausarztpraxen gelebt wird, ist mehr als nur ein Label. Sie verhindert ein Versorgungschaos (pseudo-)koordinierender digitaler Serviceleistungen, die die Hausärztin bzw. den Hausarzt umgehen oder an den Rand drängen, und letztlich die Patientinnen und Patienten in einem Wirrwarr von ungeprüften Angeboten und Daten allein ließe. Ziel muss es sein, dies zu vermeiden und stattdessen langfristig eine stabile, digital gestärkte Gesundheitsversorgung der Bevölkerung zu etablieren.

Auf dem Weg zu einer solchen hausärztlichen Medizin der Zukunft sind insbesondere die folgenden Empfehlungen zu berücksichtigen:

- Die hausärztlich zentrierte Gesundheitsversorgung der Zukunft erfordert eine weitere Stärkung der Allgemeinmedizin in der akademischen Ausbildung, der professionellen Interessenvertretung und der öffentlichen Wahrnehmung. Dies setzt eine deutliche Aufstockung der universitären Mittel entlang des Versorgungsbedarfs der Bevölkerung voraus. Gleichzeitig müssen die neuen Kompetenzen, die für die erfolgreiche Nutzung der Digitalisierung notwendig sind, in der Ausbildung vermittelt werden (Adressat: Medizinische Fakultäten, Landesministerien, Ärztekammern).
- Um die für diese Form der Gesundheitsversorgung entscheidenden hausärztlich geleiteten multiprofessionellen Teams zu ermöglichen, muss Interprofessionalität im Gesundheitswesen in der Ausbildung und Praxis der Gesundheitsberufe weiter gefördert werden, wie es etwa die neue ärztliche Approbationsordnung vorsieht (Adressat: AWMF, Medizinische Fakultäten, Ärztekammern, Landesministerien).
- Zugleich erfordert die gute und reibungslose Zusammenarbeit in multiprofessionellen Teams eine offene und faire Verständigung auf Augenhöhe über die jeweiligen Zuständigkeitsbereiche und Verantwortlichkeiten zwischen den beteiligten Berufsgruppen. Dies beinhaltet auch Lösungen im Bereich der Delegation, die belegbar dem Patientenwohl zugutekommen (Adressat: Bundesministerium für Gesundheit, Ärztekammer, Kassenärztliche Vereinigung).
- Die flächendeckende und bedarfsgerechte Versorgung schließt zudem eine stärkere Kooperation zwischen Haus- und Fachärzten bzw. -ärztinnen mit ein. Hierfür sind entsprechende regionale und digitale Kommunikationsstrukturen (wie z. B. Telekonsile) zu schaffen. Zudem sollte eine optimale Vernetzung zwischen ambulantem und stationärem Sektor dazu beitragen, spezialistische Expertise sektorenübergreifend nutzbar zu machen. Dies schließt den datenschutzkonformen Austausch von Patientendaten mit ein (Adressat: Bundesministerium für Gesundheit, Landesministerien für Gesundheit, Kassenärztliche Vereinigung, Krankenhausgesellschaft).
- Um eine umfassende holistisch-patientenorientierte Versorgung im Rahmen eines hausärztlich zentrierten Gesundheitswesens zu ermöglichen, muss die Geltendmachung von Leistungsansprüchen gegenüber den Krankenversicherungen weitgehend entbürokratisiert werden (z. B. mit Blick auf digitale Hilfsmittel im Bereich der Prävention und Rehabilitation) (Adressat: Gemeinsamer Bundesauschuss, Kassenärztliche Bundesvereinigung, Spitzenverband der Gesetzliche Krankenversicherungen).
- Die hausärztlich zentrierte Gesundheitsversorgung muss mit einer Stärkung der Gesundheitskompetenz von Bürgerinnen und Bürgern einhergehen, insbesondere durch schulischen Gesundheitsunterricht, hausärztliche Aufklärungsarbeit und Shared Decision Making, Selbsthilfe, unabhängige Informationsstellen und -portale (z. B. Nationales Gesundheitsportal; siehe dazu 8 ), digitale Gesundheitsanwendungen sowie guten Wissenschaftsjournalismus (Adressat: Bundesministerium für Bildung und Forschung, Landesministerien für Bildung, Redaktionen, Gemeinsamer Bundesausschuss, Gesetzliche Krankenversicherung, Hausärzteverband und DEGAM).
- Darüber hinaus bedarf es der weiteren digitalen Vernetzung, um Daten aus der hausärztlichen Versorgung für die Forschung verfügbar zu machen und den Wissenstransfer aus der medizinischen Forschung in die hausärztliche Praxis zu optimieren (Adressat: IT-Hersteller von Praxisinformationssystemen, Landesministerien für Bildung und Forschung und Bundesministerium für Gesundheit, Medizinische Fakultäten, Kassenärztliche Bundesvereinigung, DEGAM).
- Um die technischen Voraussetzungen für die hausärztlich zentrierte Gesundheitsversorgung der Zukunft zu schaffen, sind zentrale technische Infrastrukturen und Serviceprovider sowie datenschutzkonforme und interoperable Mechanismen für die Anonymisierung und Qualitätssicherung von Patientendaten für maschinelles Lernen notwendig. Risiken der Digitalisierung (wie z. B. Algorithmic Bias, Alert Fatigue, Überinformation) sollten minimiert werden. (Adressat: Bundesministerium für Bildung und Forschung sowie Bundesministerium für Gesundheit und Bundesministerium für Verkehr und digitale Infrastruktur, Telematik-Infrastruktur, IT-Hersteller von Praxisinformationssystemen).
- Methoden der Künstlichen Intelligenz sollten insbesondere weiterentwickelt und genutzt werden können, um Abrechnungsprozesse zur entbürokratisieren, Dokumentation effizienter zu gestalten und Rechtssicherheit zu gewährleisten. Bei standardisierten Untersuchungsverfahren (wie z. B. der Radiologie oder der Dermatologie) können Methoden der Künstlichen Intelligenz unterstützen (Adressat: Bundesministerium für Bildung und Forschung, Bundesministerium für Gesundheit, Ärztekammer, Kassenärztliche Vereinigung, gemeinsamer Bundesausschuss, Medizinische Fakultäten, Berufsverbände und medizinische Fachgesellschaften, AWMF).
- Um den Austausch großer Mengen sensibler (personenbezogener) Daten und die digitale Vernetzung verantwortungsvoll zu gestalten, sollten patientenorientierte und durch ärztliche Schweigepflicht abgesicherte Formen des Datenschutzes und Datenaustauschs entwickelt werden. Dabei ist auch auf die unkomplizierte Nutzbarkeit dieser Werkzeuge zu achten, um die Mehraufwände gering zu halten. (Adressat: Telematik-Infrastruktur, Datenschutzbeauftragte, Praxissoftwarehersteller, Kassenärztliche Vereinigung).
- Im hausärztlich zentrierten Gesundheitswesen der Zukunft müssen Fehlanreize beseitigt und Vergütungsstrukturen angepasst werden, um die wirtschaftliche Existenz der hausärztlichen Praxis und damit die entsprechende Grundversorgung sicherzustellen, sodass ärztliches Handeln ohne wirtschaftliche Abhängigkeiten ermöglicht wird (siehe 9 ) (Adressat: Bundesministerium für Gesundheit, Kassenärztliche Vereinigungen, Gemeinsamer Bundesausschuss, Spitzenverband der gesetzlichen Krankenkassen, Bundesverband der privaten Krankenkassen).
- Um eine gesamtgesellschaftliche Verständigung über die wesentlichen Ziele der solidarischen Gesundheitsversorgung zu fördern, sollten die Organisation und Vertretung von Interessen im Rahmen von Berufsverbänden sowie Patientenvereinigungen gestärkt und inklusive Verfahren öffentlicher Deliberation implementiert werden (Adressat: Berufsverbände wie Hausärzteverband und DEGAM, Patientenvereinigungen, Bundesministerium für Gesundheit).

In der Umsetzung dieser Impulse liegt aus unserer Sicht die reale Chance, sich dem Best-Case-Szenario für die Gesundheitsversorgung in Deutschland anzunähern und damit langfristig für eine zukunftsfähige Lösung zu sorgen.
